# Risk factors associated with falls in older adults: a cross-sectional study

**DOI:** 10.1590/0034-7167-2024-0455

**Published:** 2026-11-06

**Authors:** Vanessa Vieira da Motta, Pedro Miguel dos Santos Dinis Parreira, Herica Silva Dutra, André Luiz Silva Alvim, Glauce Pereira do Nascimento, Kelli Borges dos Santos, Elenir Pereira de Paiva

**Affiliations:** I Universidade Federal de Juiz de Fora. Juiz de Fora, Minas Gerais, Brazil; II Universidade de Coimbra, Escola Superior de Enfermagem. Coimbra, Portugal

**Keywords:** Accidental Falls, Aged, Risk Factors, Nursing, Health of the Elderly., Accidentes por Caídas, Anciano, Factores de Riesgo, Enfermería, Salud del Anciano.

## Abstract

**Objectives:**

to identify and correlate risk factors for falls in community-dwelling older adults.

**Methods:**

a cross-sectional epidemiological study was conducted with 200 older residents within the catchment area of a Basic Health Unit participating in the Family Health Strategy. The following instruments were used: Performance-Oriented Mobility Assessment II, Mini-Mental State Examination, Edmonton Frail Scale, Katz Index of Independence in Activities of Daily Living, and Snellen Chart.

**Results:**

falls were identified in 65% of older adults. The factors that statistically associated with the outcome were sex (p=0.047), age (p=0.001), number of comorbidities (p=0.000), balance and gait (p=0.001), cognitive impairment (p=0.009), frailty (p=0.000), activities of daily living (p=0.002), and distance vision (p=0.001).

**Conclusions:**

the main risk factors for falls in older adults include sex, advanced age, multiple comorbidities, impaired balance and gait, frailty, limitations in activities of daily living, and reduced distance visual acuity.

## RESUMEN

**Objetivos:**

identificar y correlacionar factores de riesgo de caídas en ancianos que viven en la comunidad.

**Métodos:**

estudio epidemiológico transversal con 200 residentes mayores dentro del área de influencia de una Unidad Básica de Salud que participa en la Estrategia de Salud Familiar. Se utilizaron los siguientes instrumentos: *Performance-Oriented Mobility Assessment* II, *Mini Mental State Examination, Edmonton Frail Scale, Katz Index of Independence in Activities of Daily Living* y *Snellen Chart*.

**Resultados:**

se identificaron caídas en el 65% de los ancianos. Los factores que se correlacionaron estadísticamente con el resultado fueron el sexo (p=0,047), la edad (p=0,001), el número de comorbilidades (p=0,000), el equilibrio y la marcha (p=0,001), el deterioro cognitivo (p=0,009), la fragilidad (p=0,000), las actividades básicas de la vida diaria (p=0,002), y la visión de lejos (p=0,001).

**Conclusiones:**

los principales factores de riesgo de caídas en los ancianos incluyen el sexo, la edad avanzada, las múltiples comorbilidades, el deterioro del equilibrio y la marcha, la fragilidad, las limitaciones en las actividades de la vida diaria, y la disminución de la agudeza visual de lejos.

## INTRODUCTION

Population aging is an increasingly prominent phenomenon, especially after the 1970s, resulting from the gradual decline in fertility rates and the increase in life expectancy. The aging index in Brazil in 2022, according to data from the Brazilian Institute of Geography and Statistics, reached 80.0, indicating a ratio of 80 older adults aged over 60 years for every 100 children under 15 years. There is a projection that the number of older adults will exceed the number of children by 2030 in Brazil^(1,2)^.

Given this context, it is essential to incorporate measures for preventing harm and improving the quality of life of older adults into the public policy agenda. For these measures to be effective, the demographic and epidemiological transition process must be considered, with actions promoting capabilities and well-being, seeking autonomy and independence for older adults, among others^(3,4)^.

In this context, the aging process brings frailty to older adults, making them prone to falls. However, age alone does not imply dependence and loss of autonomy. Each individual will experience the aging process in their own way, which may mean a greater or lesser need for long-term medical and social care. Falls, in turn, are unintentional events in which an individual displaces their body to a level lower than the initial one, being one of the most prevalent injuries in older adults. As a result of falls, older adults may experience, among other consequences, loss of independence for daily activities, reduced functional capacity, and social isolation^(5)^.

The risk factors that lead to falls are multiple, and are classified as intrinsic and extrinsic. Intrinsic factors are those that are not modifiable, such as sex, age, comorbidities, physiological changes, reduced cognitive capacity, previous falls, and gait disorders. Extrinsic factors are related to the environment and behavioral factors, such as fast gait, climbing on chairs to reach objects, slippery floors, low lighting, and loose rugs on the floor^(6,7)^.

Researchers in the United Kingdom highlighted that advanced age was associated with a higher risk of falls in both sexes. Among women, the risks were 1.03 in the presence of depressive symptoms and 1.12 in cases of incontinence. In men, the risks increased to 1.04 with a greater number of comorbidities, to 1.10 with high levels of pain, and to 1.23 with poorer balance^(5)^. From another perspective, a systematic review and meta-analysis investigated differences in the prevalence of falls among ethnic groups in community-dwelling older adults, showing that the Asian group had the lowest prevalence (13.89%), followed by the Hispanic and black groups, with prevalences of 18.54% and 18.60%, respectively. The white group had the highest prevalence of 23.77%, showing persistent differences even after adjusting for other risk factors^(6)^.

Policies aimed at preventing and promoting care related to falls in older adults seek to establish instruments that can investigate the causes of falls, as well as develop plans to intervene in the identified scenarios. For the planning and execution of efficient preventive actions, it is fundamental to identify vulnerable groups, aiming to ensure the preservation of older adults’ functionality and well-being^(8)^.

In this regard, nursing professionals play a fundamental role in improving the quality of life of older adults through specific care, encompassing the assessment of risk factors for falls and the development of educational practices aimed at preventing this adverse event^(9)^.

By understanding the risk factors involved in falls, nursing professionals can use this knowledge in their practice, promoting actions at the individual, social, and community levels, bringing benefits to this specific population^(10)^.

Despite the high rate of falls among older adults, research into risk factors and the impacts on health and society, along with strategies for preventing these incidents, is still in its early stages. Therefore, the importance of multidimensional research encompassing areas of health education, professional training, and the development of safer workplaces is highlighted.

Given the above, the following questions arose: what are the risk factors for falls in older adults living in the community? What is the correlation between risk factors and falls?

## OBJECTIVES

To identify and correlate risk factors for falls in community-dwelling older adults of a major city in the *Zona da Mata Mineira*.

## METHODS

### Ethical aspects

This study complied with the ethical and legal requirements for research involving human subjects. Participants were coded to ensure confidentiality. Prior to data collection, participants were informed about the study and signed an Informed Consent Form. Approval was obtained from the Research Ethics Committee of the *Universidade Federal de Juiz de Fora*. The research risks are considered minimal, since the methods and techniques used during data collection did not include interventions on participants. The researchers were careful to use communication, relational, and human respect techniques to make the interview pleasant and respectful.

### Study design, period, and setting

This is a cross-sectional epidemiological study, guided by the STrengthening the Reporting of OBservational studies in Epidemiology protocol. Data were collected from April to September 2019 through home visits. The research was conducted in Juiz de Fora, Minas Gerais, in the area covered by a Basic Health Unit (BHU) adhering to the Family Health Strategy (FHS), registered in the electronic system of the Brazilian Unified Health System: e-SUS. The research site had two FHS teams, divided into ten micro-areas, and one uncovered area.

### Population or sample; inclusion and exclusion criteria

Through the e-SUS system, the total population of older adults in the coverage area was identified as 1,536, comprising 641 men and 895 women. After calculating the sample size for this population, with a 95% confidence level and a 5% margin of error, the estimated sample size was 308 participants. Data on eligible older adults (name and address) were collected from primary healthcare professionals via e-SUS, allowing for blind selection of participants through simple random sampling. Of the 308 selected participants, 108 were lost due to the following reasons: older adults were working (37); address not found (29); no longer residing at the registered address (14); refusal to participate (21); and hospitalization (7). As there was no provision for replacing lost participants, the final sample consisted of 200 participants.

Individuals over 60 years of age, residing in the selected BHU coverage area, who agreed to voluntarily participate in the research and were able to walk were included in the study. Older adults who did not respond to the call at their residence on more than two attempts on different days were excluded.

### Study protocol

In order to achieve the objectives of this research, five instruments were applied to assess the risk factors for falls to which older adults are exposed, namely: Performance-Oriented Mobility Assessment II^(11)^, to assess static and dynamic balance and mobility; Mini Mental State Examination^(12)^, to assess cognitive ability; Edmonton Frail Scale^(13)^, to assess frailty; and Katz Index of Independence in Activities of Daily Living^(14)^ and Snellen Chart^(15)^, to assess distance visual acuity. Finally, a semi-structured interview instrument, developed by the researcher herself, was applied to characterize participants, containing variables such as age, sex, lifestyle habits, family income, education level, comorbidities, and fall events.

### Analysis of results and statistics

Data were organized in a spreadsheet using Microsoft Excel for Windows and subsequently statistically analyzed using the Statistical Package for the Social Sciences software version 22.0, considering a 95% level of evidence, treated with descriptive and correlational statistics.

Descriptive analysis was performed using absolute frequency (n), relative frequency (%), mean (µ), and standard deviation (δ). To perform the bivariate analysis, the normality of quantitative variables was first investigated using the Kolmogorov-Smirnov normality test. The dependent variable used was the occurrence of falls.

Statistical tests were performed based on the characteristics of the variables to be analyzed. Pearson’s chi-square test and Fisher’s exact test were used to analyze the association between nominal and ordinal variables. Spearman’s correlation was used to analyze quantitative variables. The dependent variable was “fall”, analyzed using a binary model (yes or no) when associated with categorical data and numerically by the number of falls self-reported by older adults when associated with numerical data.

## RESULTS

The mean number of falls was 3.08, with a standard deviation of 3.02. The maximum number of falls per person was 15. Of those who fell, 50.8% reported more than one fall event, and 65% of older adults reported having fallen at least once.

The sample consisted of 200 older adults, 63.5% of whom were female. The minimum age was 60 years, and the maximum was 96 years. Most older adults were not oldest-old (75.5%), whereas those aged over 80 years accounted for 24.5%. The mean age was 73.69 (±9.18), with a mode of 62 years. Most older adults were white (70.5%), had primary education (63.5%), were Catholic (78%), had a monthly income between one and three minimum wages (51.5%), were married or living in a stable union (50.5%), and did not live alone (85%).

Concerning lifestyle, the majority were non-smokers (58.5%). Additionally, 31% were former smokers, 8.5% were current smokers, and 2% were passive smokers. Most presented some type of comorbidity (78%). Of the participants with comorbidities, 54.5% had more than one pathology, and one reported five or more. They reported a prevalence of diseases such as hypertension (59.5%) and diabetes mellitus (25%). Only 14% used some type of gait aid, such as a walker or cane.

Sex (p=0.047) and arthritis (p=0.036) showed statistical significance with the fall event. Female sex increased the chance of falls by 1.828 times (CI 1.005-3.325), and the presence of arthritis increased the chance of falling by 4.435 times (CI 1.169-13.268). In the quantitative analysis, Spearman’s correlation was performed, which showed a positive correlation between the number of falls and age (p=0.001; rho=0.226), and falls and the number of comorbidities (p<0.001; rho=0.248).

In correlational analysis, balance showed a negative correlation with falls. The lower the balance, the greater the number of falls (p=0.001; rho=-0.236).

In static balance, the occurrence of falls was associated with the inability to maintain stability with feet side by side (p=0.045), balancing with the support of only one leg (p=0.006), in a semi-tandem position (p=0.004) or tandem position (p=0.001), and on tiptoes (p=0.026).

In dynamic balance, when standing up, on average, 86.4% of older adults who presented as unable or unstable experienced at least one fall (p=0.026). Older adults unable to control their body from a sitting to a lying position (p=0.012) and unstable during compensatory balance (p=0.003) also fell more often.

It was also found that older adults who presented with missteps (p=0.019) and had difficulty stepping over objects on the floor (p=0.038) had a higher prevalence of falls.

To assess participants’ cognitive deficit, their level of education was considered. The majority (95%) did not have a cognitive deficit, according to the cut-off point of the instrument used. The maximum score on the scale was 30 points, and participants presented a mean score of 25.73 (±4.33). In bivariate analysis, there was a significant negative correlation, such that the lower the cognitive ability, the greater the number of falls reported (p=0.009; rho=-0.186).

To assess frailty on the Edmonton Frailty Scale, the higher the score, the greater the frailty of older adults, with a maximum score of 17. The lowest score obtained was zero, and the highest was ten, indicating that no participant presented severe frailty (>11). The mean score was 4.31 (±2.49). Table 1 shows that more than half of participants (54%) did not have frailty; 24% were apparently vulnerable; 16.5% had mild frailty; and 5.5% had moderate frailty. In the bivariate analysis, frailty and falls showed a positive correlation, and the greater the frailty of older adults, the greater the number of falls (p<0.001; rho=0.312).

**Table 1:** Frailty and its association with falls in the municipality of *Zona da Mata Mineira* (N=200), Juiz de Fora, Minas Gerais, Brazil, 2019

Frailty	Frequency	Yes	No	*p* value
**Cognition (clock drawing)**				
Approved	59 (29.5)	38 (64.4)	21 (35.6)	0.993
Minimal errors	63 (31.5)	41 (65.1)	22 (34.9)	
Failed	78 (39)	51 (65.4)	27 (34.6)	
**Hospitalization in the last 12 months**				
None	156 (77.9)	95 (60.9)	61 (39.1)	
1-2 times	42 (21.1)	34 (81)	8 (19)	**0.046**
>2 times	2 (1)	1 (50)	1 (50)	
**How is health**				
Excellent, very good or good	136 (68.3)	83 (61)	53 (39)	
Average	60 (30.2)	43 (71.7)	17 (28.3)	0.156
Poor	3 (1.5)	3 (100)	0 (0)	
**How many daily activities they need help with**				
0-1	150 (75.4)	94 (62.7)	56 (37.3)	
2-4	30 (15.1)	19 (63.3)	11 (36.7)	0.177
5-8	19 (9.5)	16 (12.4)	3 (4.3)	
**Have social support**				
Always	178 (89.4)	113 (63.5)	65 (36.5)	
Sometimes	16 (8)	13 (81.3)	3 (18.8)	0.353
Never	5 (2.5)	3 (60)	2 (40)	
**Use more than five medications**				
Yes	80 (40)	58 (72.5)	22 (27.5)	0.135
No	120 (60)	72 (60)	48 (40)	
**Forget to take medications**				
Yes	28 (14.1)	20 (71.4)	8 (28.6)	0.430
No	171 (85.9)	109 (63.7)	62 (36.3)	
**Lost weight recently**				
Yes	61 (30.7)	42 (68.9)	19 (31.1)	0.429
No	138 (69.3)	87 (63)	51 (37)	
**Often feel sad**				
Yes	33 (16.6)	23 (69.3)	10 (30.3)	0.331
No	165 (82.9)	106 (64.2)	59 (35.8)	
**Have urinary incontinence**				
Yes	28 (14.1)	22 (78.6)	6 (21.4)	0.100
No	171 (85.9)	107 (62.6)	64 (37.4)	
**Travel time (three meters)**				
0-10 seconds	56 (28)	29 (51.8)	27 (48.2)	
11-20 seconds	86 (43)	55 (42.3)	31 (44.3)	**0.008**
>20 seconds	58 (29)	46 (79.3)	12 (20.7)	

Assessing the aspects that indicate frailty, two showed a statistical association: hospitalization in the last 12 months and functional performance. An association was found between falls and the number of hospitalizations in the last 12 months (p=0.046), with 81% of older adults who were hospitalized one or two times during this period experiencing a fall.

Functional performance was assessed using the timed “stand and walk” test, in which the participant had to stand up from a chair, walk three meters, and return to the starting point, sitting down again. In this assessment, Table 2 shows that there was a positive statistical correlation with the number of falls, and the longer the gait time, the greater the number of falls (p=0.008; rho=0.195). The chi-square test also showed an association with falls (p=0.008).

**Table 2:** Activities of daily living and their association with falls (N=200), Juiz de Fora, Minas Gerais, Brazil, 2019

Activity		Falls n (%)		*p* value
	Frequency	Yes	No	
**Bathing**				
Independent	185 (92.5)	116 (62.7)	69 (37.3)	
Some dependency	11 (5.5)	11 (100)	0 (0)	**0.038**
Dependent	4 (2)	3 (75)	1 (25)	
**Dressing**				
Independent	188 (94)	120 (63.8)	68 (36.2)	
Some dependency	4 (2)	4 (100)	0 (0)	0.270
Dependent	8 (4)	6 (75)	2 (25)	
**Personal hygiene**				
Independent	193 (96.5)	124 (64.2)	69 (35.8)	
Some dependency	6 (3)	6 (100)	0 (0)	0.077
Dependent	1 (0.5)	0 (0)	1 (100)	
**Sitting down, lying down, and standing up**				
Independent	191 (95.5)	121 (63.4)	70 (36.6)	0.028^*^
Some dependency	9 (4.5)	9 (100)	0 (0)	
Dependent	0 (0)	0 (0)	0 (0)	
**Sphincter control**				
Complete	176 (88)	111 (63.1)	65 (36.9)	
Occasional accidents	21 (10.5)	16 (76.2)	5 (23.8)	0.217
Incontinent	3 (1.5)	3 (100)	0 (0)	
**Nutrition**				
Independent	197 (100)	128 (65)	69 (35)	
Some dependency	3 (1.5)	2 (66.7)	1 (33.3)	0.951
Dependent	0 (0)	0 (0)	0 (0)	

* *p value calculated using Fisher’s exact test.*

In bivariate analysis, there was a statistical correlation with falls, and the greater the number of falls, the lower the independence (p=0.002; rho=-0.220). The basic activities that had a statistical association with falls were bathing (p=0.038) and the ability to sit, lie down, and stand (p=0.028). Of the participants who showed some dependence in these two activities, all had experienced an episode of falls (Table 3).

**Table 3:** Visual capacity and its association with falls in older adults (N=200), Juiz de Fora, Minas Gerais, Brazil, 2019

Visual capacity		Falls n (%)				95%CI	
	Frequency	Yes	No	*p* value	OR	Lower	Upper
**Distance vision**							
Satisfactory	116 (58)	86 (74.1)	30 (25.9)	**0.001**	2.606	1.435	4.733
With some loss	84 (42)	44 (52.4)	40 (47.6)				
**Near vision**							
Satisfactory	132 (68)	88 (66.7)	44 (33.3)	0.464	-		
With some loss	62 (32)	38 (61.3)	24 (38.7)				

*95%CI - 95% Confidence Interval; OR - Odds Ratio.*

In distance visual acuity assessment, 58% of older adults had satisfactory vision, and 42% had moderate to severe vision loss. In bivariate analysis, distance visual acuity showed significance when associated with falls (p=0.001). Spearman’s correlation confirmed this finding, where the greater the vision loss, the greater the number of falls (p<0.001; rho=-0.248). Older adults with impaired distance visual acuity had a 2.606 times greater chance of falling (CI 1.435 - 4.733).

## DISCUSSION

The sample showed a feminization, composed mostly of white individuals with incomplete primary education, Catholic, married or in a stable union, with a monthly income between one and three minimum wages, and living with family members. A similar study identified that factors such as female gender, low education level, and widowhood are related to people with vulnerability^(16)^. It is known that, at this stage of life, family support is essential in maintaining the physical and psychological well-being of older adults, who accumulate losses due to old age that are often difficult to cope with. This phase of life requires specific care, emphasizing respect and the promotion of autonomy, cultivating a support network, as this is the path to achieving future scenarios of appreciation for this population^(17)^.

Analyzing the occurrence of falls, 65% of older adults reported having fallen at least once; of these, half reported more than one fall event. The occurrence and recurrence of falls can have significant consequences for older adults, such as dependence, social isolation, and loss of functional capacity^(18)^. A study identified that among the most prevalent consequences in older adults after a fall are abrasions, fractures, and sprains. Furthermore, permanent consequences are present in a large proportion of older adults, such as fear of another fall, impaired gait ability, increased dependence in daily activities with loss of autonomy, and the onset or worsening of depression^(19)^. A study that used game design for fall prevention showed that the development of an innovative game generated significant impacts in reducing this problem^(20)^.

The sample identified that 14% of older adults used devices such as walkers and canes. This finding was also observed in a similar study with 214 older adults living in the community, in which the prevalence of gait aid use was 12.7%^(21)^. Furthermore, physical impairments resulting from aging, such as changes in posture and balance, reduce gait efficiency in older adults, and the use of assistive devices may be indicated in these cases^(22)^.

A significant relationship between falls and cognitive impairment stands out in the sample, since the lower the cognitive ability, the greater the number of falls. Supporting this finding, another study, which analyzed the occurrence of falls, found that cognitive impairment maintained a statistically significant association with this event^(23)^. It is known that cognitive decline is a consequence of the aging process and is related to deficiencies in concentration and attention span in older adults. This can also cause short-term memory impairment and lead to distractibility, which can contribute to exposure to fall risk situations such as tripping^(24)^. A previous study, conducted in Brazil, showed that frailty is associated with lower levels and more pronounced declines in cognitive function, with stronger associations for executive function^(23)^.

When analyzing frailty, a correlation with falls was found (p<0.001), such that the frailer the older adult, the more prone to falls they are. A similar result was found in another study conducted with older adults in which frailty syndrome was related to the occurrence of falls (p=0.001)^(25)^. Moreover, it was found that more than half of participants did not present with frailty, while another 46% presented with frailty to varying degrees. The study showed a high prevalence of frailty in older adults and also highlighted the relationship between frailty and sex, age, education level, and motor manifestations^(26)^.

As for older adults’ travel time, it was found to be related to the occurrence of falls, with 77.7% of older adults who fell showing a reduction in gait speed. Reduced gait speed is one of the factors that define the presence of frailty and has been observed in 100% of the oldest-old and in 80% of frail older adults in other studies involving this population^(27)^.

Falls in older adults can signal the onset of frailty or the appearance of acute illness. Of the older adults who experienced a fall, 26.9% had been hospitalized at least once in the past year. These events were statistically associated (p=0.046). Another study corroborates this finding, in which 25.3% of those who fell had been hospitalized during the same period (p=0.001)^(28)^.

The association between falls and hospitalization, although common, was not widely found in the sample, highlighting the importance of conducting research that addresses this relationship more deeply. It is known that frailty in older adults makes them more prone to health problems, which can increase the need for hospitalization. After a fall, frail older adults require a prolonged period for recovery and rehabilitation, which can increase the length of hospital stay and consequently the financial costs for the system and the individual. Authors have shown that fall risk screening should consider the order and timing of the onset of physical frailty and cognitive impairment^(27,28)^.

Regarding activities of daily living, it was found that increased dependence in these activities was related to an increased number of falls (p=0.002; rho=-0.220). Research showed that dependence in basic activities is associated with falls (p=0.049). However, older adults with functional limitations may present with a lack of balance or motor coordination, which makes it more difficult to perform daily activities. On the other hand, functional independence in older adults, particularly among the oldest-old, was associated with the absence of one of the main consequences of falls: hip fracture (p=0.042)^(29)^.

In the analysis of distance visual acuity, 42% of older adults presented some loss, a fact associated with the occurrence of falls (p=0.001). It was also found that the lower the distance visual acuity, the greater the occurrence of falls (p<0.001; rho=-0.248). Visual impairment in older adults can lead to difficulties in maintaining postural control, exposing these individuals to falls. On the other hand, a study showed that older adults with reduced visual acuity were 1.96 times more likely to suffer a fall^(30)^. Furthermore, the sample identified that one-third of older adults presented with visual impairment, while 14% reported hearing loss. The authors identified that sensory losses are an important factor, as they compromise functional capacity and therefore affect the quality of life of older adults, decreasing their functional capacity and making them less active^(31)^.

### Study limitations

The cross-sectional design of the study is a limitation, as it does not allow for the assessment of causal relationships between variables. Furthermore, the sample consisted of older adults from a specific area, with a defined socioeconomic profile and level of education, which may limit the generalizability of the findings. Another relevant limitation is the possible influence of participants’ memory, which may compromise the accuracy of self-reported information about falls and other retrospective events.

### Contributions to nursing, health, or public policy

This study makes a significant contribution to the field of nursing by highlighting factors that expose older adults to falls, as well as strengthening professionals’ autonomy in implementing safe and low-cost preventive interventions. In particular, it emphasizes the early identification of fall risk and the application of assessment instruments by nurses, which can be carried out with older adults treated in Primary Care as well as in long-term care facilities. We consider this a viable intervention that can be widely available and adapted to different institutional contexts.

## CONCLUSIONS

Factors identified as the strongest predictors of fall risk were sex, age, number of comorbidities, balance and gait, frailty, activities of daily living, and reduced distance visual acuity. The recognition of the main risk factors for falls in older adults, evidenced in this study, contributes to the reorientation of care practices, especially in Primary Care. In this way, the healthcare team can direct its practice based on evidence and carry out its actions preventively for individuals at higher risk. A safer and more qualified care practice enables a reduction in the incidence of falls and their complications.

## Data Availability

The research data are available within the article.
