# Supplementary material for: Risk factors associated with falls in older adults: a cross-sectional study
Source: Rev Bras Enferm. 2026 Nov 6;79:e20240455. doi: 10.1590/0034-7167-2024-0455 (PMC13742976; doi:10.1590/0034-7167-2024-0455)
Supplement: Translation

# Translation: Fatores de risco correlacionados às quedas em idosos: estudo transversal

**DOI:** 10.1590/0034-7167-2024-0455pt

**Authors:** Vanessa Vieira da Motta, Pedro Miguel dos Santos Dinis Parreira, Herica Silva Dutra, André Luiz Silva Alvim, Glauce Pereira do Nascimento, Kelli Borges dos Santos, Elenir Pereira de Paiva

**Affiliations:** I Universidade Federal de Juiz de Fora. Juiz de Fora, Minas Gerais, Brasil; II Universidade de Coimbra, Escola Superior de Enfermagem. Coimbra, Portugal

**Keywords:** Acidentes por Quedas, Idoso, Fatores de Risco, Enfermagem, Saúde do Idoso.

## Abstract

**Objetivos:**

identificar e correlacionar os fatores de risco às quedas em idosos residentes de comunidade.

**Métodos:**

estudo epidemiológico de delineamento transversal, realizado com 200 idosos residentes na área de abrangência de uma Unidade Básica de Saúde participante da Estratégia Saúde da Família. Utilizaram-se os seguintes instrumentos: *Performance-Oriented Mobility Assessment* II, *Mini Mental State Examination, Edmonton Frail Scale, Katz Index of Independence in Activities of Daily Living* e *Snellen Chart*.

**Resultados:**

identificou-se queda em 65% dos idosos. Os fatores que se correlacionaram estatisticamente ao desfecho foram sexo (p=0,047), idade (p=0,001), número de comorbidades (p=0,000), equilíbrio e marcha (p=0,001), déficit cognitivo (p=0,009), fragilidade (p=0,000), atividades básicas diárias (p=0,002), e visão de longe (p=0,001).

**Conclusões:**

os principais fatores de risco para quedas em idosos incluem sexo, idade avançada, múltiplas comorbidades, comprometimento do equilíbrio e da marcha, fragilidade, limitações nas atividades diárias, e redução da acuidade visual para longe.

## INTRODUÇÃO

O envelhecimento populacional é um evento que tem se tornado cada vez mais emergente, especialmente após a década de 1970, decorrente da gradativa queda da taxa de fecundidade e do aumento da expectativa de vida. O índice de envelhecimento no Brasil em 2022, de acordo com os dados do Instituto Brasileiro de Geografia e Estatística, chegou a 80,0, indicando um quantitativo de 80 idosos maiores de 60 anos para cada cem crianças menores de 15 anos. Há uma projeção de que o número de idosos ultrapasse o número de crianças até 2030 no Brasil^(1,2)^.

Diante desse contexto, torna-se imprescindível a incorporação, na agenda das políticas públicas, de medidas de prevenção de agravos e melhoria da qualidade de vida da pessoa idosa. Para a efetividade dessas medidas, deve-se levar em consideração o processo de transição demográfica e epidemiológica, com ações de promoção de capacidades e bem-estar, busca da autonomia e independência do idoso, entre outras^(3,4)^.

Nesse contexto, o processo de envelhecimento traz ao idoso fragilidade, o que o torna propenso ao evento de queda. Contudo, a idade por si só não pressupõe dependência e perda da autonomia. Cada indivíduo viverá o processo de envelhecimento de uma forma, o que poderá significar maior ou menor necessidade de cuidados médicos e sociais a longo prazo. As quedas, por sua vez, são eventos não intencionais em que o indivíduo desloca seu corpo a um nível inferior ao inicial, sendo um dos agravos mais prevalentes na população idosa. Em decorrência das mesmas, o idoso pode apresentar, entre outras consequências, perda da independência para atividades diárias, redução da capacidade funcional e isolamento social^(5)^.

Os fatores de risco que levam às quedas são múltiplos, sendo classificados em intrínsecos e extrínsecos. São considerados intrínsecos aqueles não modificáveis, como sexo, idade, comorbidades, alterações fisiológicas, redução da capacidade cognitiva, quedas anteriores e distúrbios de marcha. E os extrínsecos são relacionados ao meio ambiente e aos fatores comportamentais, como marcha acelerada, subida em cadeiras para alcançar objetos, pisos escorregadios, baixa iluminação e tapetes soltos no chão^(6,7)^.

Pesquisadores do Reino Unido destacaram que a idade avançada foi associada a um maior risco de quedas em ambos os sexos. Entre as mulheres, os riscos foram de 1,03 em presença de sintomas depressivos e de 1,12 em casos de incontinência. Nos homens, os riscos aumentaram para 1,04 com maior número de comorbidades, para 1,10 em níveis elevados de dor e para 1,23 diante de pior equilíbrio^(5)^. Em outra perspectiva, estudo de revisão sistemática e meta-análise investigou diferenças na prevalência de quedas entre grupos étnicos em idosos comunitários, evidenciando que o grupo asiático apresentou a menor prevalência (13,89%), seguido pelos grupos hispânico e negro, com prevalências de 18,54% e 18,60%, respectivamente. O grupo branco teve a maior prevalência de 23,77%, evidenciando diferenças persistentes mesmo após ajuste para outros fatores de risco^(6)^.

As políticas voltadas para prevenir e promover cuidados relacionados às quedas na população idosa têm como propósito instituir instrumentos que possam averiguar motivos que as desencadeiam, tal como elaborar planos para intervir nos cenários identificados. Para o planejamento e a execução de ações preventivas eficientes, é fundamental identificar os grupos vulneráveis, visando assegurar a preservação da funcionalidade e do bem-estar da população idosa^(8)^.

Nesse sentido, o profissional enfermeiro exerce um papel fundamental para proporcionar uma melhoria na qualidade de vida da pessoa idosa, através de cuidados específicos, que abarcam a avaliação dos fatores de risco para quedas e o desenvolvimento de práticas educativas direcionadas à prevenção desse evento adverso^(9)^.

Ao compreender os fatores de risco envolvidos na ocorrência de quedas, a enfermagem é capaz de utilizar esses conhecimentos na prática profissional, promovendo ações em nível individual, social e comunitário, trazendo benefícios a essa população específica^(10)^.

Apesar do índice elevado de quedas em pessoas idosas, a investigação dos fatores de risco e dos impactos gerados à saúde e à sociedade, juntamente com as estratégias de prevenção do incidente, até o momento, ainda é incipiente. Dessa maneira, salienta-se a relevância de investigação multidimensional que englobe áreas de educação em saúde, capacitação dos profissionais e desenvolvimento de locais mais seguros.

Diante do exposto, surgiram as seguintes questões: quais os fatores de risco para queda em idosos residentes na comunidade? Qual a correlação entre fatores de risco e quedas?

## OBJETIVOS

Identificar e correlacionar os fatores de risco para quedas em idosos residentes na comunidade de uma cidade polo da Zona da Mata Mineira.

## MÉTODOS

### Aspectos éticos

Este estudo atendeu aos critérios éticos e legais exigidos em pesquisas envolvendo seres humanos. Os participantes foram codificados para a garantia do sigilo. Previamente à realização da pesquisa, houve esclarecimento sobre a pesquisa e assinatura do Temo de Consentimento Livre e Esclarecido pelos participantes e pelas pesquisadoras. Foi obtida aprovação no Comitê de Ética em Pesquisa da Universidade Federal de Juiz de Fora. Os riscos da pesquisa são considerados mínimos, uma vez que os métodos e as técnicas utilizadas durante a coleta de dados não incluíram a realização de intervenções no usuário. Houve preocupação das pesquisadoras em utilizar técnicas comunicacionais, relacionais e de respeito humano, a fim de tornar a entrevista agradável e respeitosa.

### Desenho, período e local do estudo

Trata-se de estudo epidemiológico, transversal, norteado pelo protocolo *STrengthening the Reporting of OBservational studies in Epidemiology*. Os dados foram coletados no período de abril a setembro de 2019 por meio de visitas domiciliares. A pesquisa foi realizada em Juiz de Fora, Minas Gerais, na área de abrangência de uma Unidade Básica de Saúde (UBS) aderente à Estratégia Saúde da Família (ESF), com cadastro no sistema eletrônico do Sistema Único de Saúde: e-SUS. O local da pesquisa possuía duas equipes de ESF, divididas em dez microáreas, e uma área descoberta.

### População ou amostra; critérios de inclusão e exclusão

Por meio do sistema e-SUS, foi identificada a população total de idosos na área de abrangência, com 1.536 idosos, sendo 641 homens e 895 mulheres. Após o cálculo amostral para essa população, com 95% de nível de confiança e 5% de margem de erro, o tamanho estimado da amostra foi de 308 participantes. Os dados dos idosos elegíveis para a pesquisa (nome e endereço) foram captados com os profissionais da UBS no e-SUS, sendo possível, assim, fazer a seleção às cegas dos participantes da pesquisa por amostra aleatória simples. Dos 308 participantes selecionados, houve 108 perdas em razão dos seguintes motivos: idoso estar trabalhando (37); endereço não encontrado (29); não residir mais no endereço cadastrado (14); recusa em participar (21); e internação (7). Como não houve previsão de reposição de perda, a amostra final perfez 200 participantes.

Foram incluídos no estudo indivíduos com idade superior a 60 anos, residentes na área de abrangência da UBS selecionada, concordantes em participar voluntariamente da pesquisa e com capacidade de deambular. Foram excluídos os idosos que não atenderam ao chamado na residência em mais de duas tentativas em dias distintos.

### Protocolo do estudo

A fim de alcançar os objetivos desta pesquisa, foram aplicados cinco instrumentos que permitiram avaliar os fatores de risco para quedas aos quais os idosos estão expostos, sendo eles: *Performance-Oriented Mobility Assessment II* ^(11)^, para avaliação do equilíbrio estático e dinâmico e da mobilidade; *Mini Mental State Examination* ^(12)^, para avaliação da capacidade cognitiva; *Edmonton Frail Scale* ^(13)^, para avaliação da fragilidade; e *Katz Index of Independence in Activities of Daily Living* ^(14)^ e *Snellen Chart* ^(15)^, para avaliação da acuidade visual para longe. Por fim, foi aplicado um instrumento de entrevista semiestruturada para a caracterização do participante, elaborado pela própria pesquisadora, contendo variáveis como idade, sexo, hábitos de vida, renda familiar, escolaridade, comorbidades e evento de queda.

### Análise dos resultados e estatística

Os dados foram organizados em planilha no programa *Microsoft Excel for Windows* e posteriormente analisados estatisticamente no *software Statistical Package for the Social Sciences,* versão 22.0, considerando-se o nível de evidência de 95%, tratados por estatística descritiva e correlacional.

A análise descritiva foi feita através da frequência absoluta (n), relativa (%), média (µ) e desvio padrão (δ). Para realizar a análise bivariada, primeiramente, foi investigada a normalidade das variáveis quantitativas pelo teste de normalidade de Kolmogorov-Smirnov. A variável dependente utilizada foi a ocorrência de quedas.

Foram realizados os testes estatísticos a partir das características das variáveis a serem analisadas. Para a associação das variáveis nominais e ordinais, foram utilizados os testes qui-quadrado de Pearson e extrato de Fisher. Para a análise das variáveis quantitativas, foi feita a correlação de Spearman. A variável dependente foi “queda”, analisada no modelo binário (sim ou não) quando associada a dados categóricos e, de forma numérica, pela quantidade de quedas autorreferidas pelos idosos quando associada a dados numéricos.

## RESULTADOS

A média de quedas foi de 3,08, com desvio padrão de 3,02. O número máximo de quedas por pessoa foi de 15. Dos caidores, 50,8% apresentaram mais de um evento de queda, e 65% dos idosos informaram ter caído pelo menos uma vez.

A amostra foi composta por 200 idosos, sendo 63,5% do sexo feminino. A idade mínima foi de 60 anos, e a máxima, de 96 anos. A maioria dos idosos era não longeva (75,5%), enquanto aqueles com idade superior a 80 anos correspondiam a 24,5%. A idade média foi de 73,69 (±9,18), com moda de 62 anos. A maior parte dos idosos era branca (70,5%), possuía ensino fundamental (63,5%), era católica (78%), apresentava renda mensal entre um e três salários mínimos (51,5%), era casada ou vivia em união estável (50,5%) e não vivia sozinha (85%).

Em relação ao estilo de vida, a maioria era não fumante (58,5%). Além disso, 31% eram ex-fumantes, 8,5%, fumantes, e 2%, fumantes passivos. A maior parte apresentava algum tipo de comorbidade (78%). Dos participantes com comorbidades, 54,5% possuíam mais de uma patologia, e um informou cinco ou mais. Informaram prevalência de doenças como hipertensão arterial (59,5%) e diabetes mellitus (25%). Apenas 14% utilizavam algum tipo de auxílio para marcha, como andador e bengalas.

Sexo (p=0,047) e artrite (p=0,036) apresentaram significância estatística com o evento queda. O sexo feminino aumentou em 1,828 vezes (IC 1,005-3,325) a chance de ocorrência de quedas, e a presença de artrite aumentou em 4,435 vezes (IC 1,169-13,268) a chance de cair. Na análise quantitativa, foi realizada a correlação de Spearman, que se mostrou positiva entre o número de quedas e a idade (p=0,001; rho=0,226), e quedas e o número de comorbidades (p<0,001; rho=0,248).

Na análise correlacional, o equilíbrio obteve correlação negativa com as quedas. Quanto menor o equilíbrio, maior o número de quedas (p=0,001; rh0=-0,236).

No equilíbrio estático, estiveram associadas à ocorrência de quedas e à incapacidade de se manter estável com os pés lado a lado (p=0,045), equilibrando com o apoio de apenas uma perna (p=0,006), em posição de semi-tandem (p=0,004) ou tandem (p=0,001) e na ponta dos pés (p=0,026).

No equilíbrio dinâmico, no ato de se levantar, em média, 86,4% dos idosos que se apresentaram incapazes ou instáveis apresentaram ao menos um episódio de queda (p=0,026). Idosos incapazes de controlar o corpo da posição sentada para deitada (p=0,012) e instáveis durante o equilíbrio compensatório (p=0,003) também caíram mais.

Foi possível verificar ainda que idosos que apresentavam na marcha passos em falso (p=0,019) e tinham dificuldades em transpor objetos no chão (p=0,038) tinham maior prevalência de quedas.

Para avaliar o déficit cognitivo dos participantes, foi considerado o nível de escolaridade que apresentavam. A maioria (95%) não possuía déficit cognitivo, de acordo com o ponto de corte do instrumento utilizado. A pontuação máxima da escala era 30 pontos, e os participantes apresentaram pontuação média de 25,73 (±4,33). Na análise bivariada, houve correlação negativa significativa, de forma que, quanto menor a capacidade cognitiva, maior o número de quedas relatadas (p=0,009; rho=-0,186).

Para avaliar a fragilidade na *Edmonton Frailty Scale*, quanto maior a pontuação, maior a fragilidade do idoso, sendo a pontuação máxima 17. A menor pontuação obtida foi zero, e a maior foi dez, indicando que nenhum participante apresentava fragilidade severa (>11). A pontuação média foi de 4,31 (±2,49). A Tabela 1 mostra que mais da metade dos participantes (54%) não possuía fragilidade; 24% eram aparentemente vulneráveis; 16,5% tinham fragilidade leve; e 5,5% tinham fragilidade moderada. Na análise bivariada, fragilidade e queda apresentaram correlação positiva, e quanto maior a fragilidade do idoso, maior o número de quedas (p<0,001; rho=0,312).

**Tabela 1:** Fragilidade e associação com quedas no município da Zona da Mata Mineira (N=200), Juiz de Fora, Minas Gerais, Brasil, 2019

Fragilidade	Frequência	Sim	Não	Valor de *p*
**Cognição (desenho do relógio)**				
Aprovado	59 (29,5)	38 (64,4)	21 (35,6)	0,993
Erros mínimos	63 (31,5)	41 (65,1)	22 (34,9)	
Reprovado	78 (39)	51 (65,4)	27 (34,6)	
**Internação em 12 meses**				
Nenhuma	156 (77,9)	95 (60,9)	61 (39,1)	
1-2 vezes	42 (21,1)	34 (81)	8 (19)	**0,046**
>2 vezes	2 (1)	1 (50)	1 (50)	
**Como está a saúde**				
Excelente, muito boa ou boa	136 (68,3)	83 (61)	53 (39)	
Razoável	60 (30,2)	43 (71,7)	17 (28,3)	0,156
Ruim	3 (1,5)	3 (100)	0 (0)	
**Quantas atividades diárias precisa de ajuda**				
0-1	150 (75,4)	94 (62,7)	56 (37,3)	
2-4	30 (15,1)	19 (63,3)	11 (36,7)	0,177
5-8	19 (9,5)	16 (12,4)	3 (4,3)	
**Tem suporte social**				
Sempre	178 (89,4)	113 (63,5)	65 (36,5)	
Algumas vezes	16 (8)	13 (81,3)	3 (18,8)	0,353
Nunca	5 (2,5)	3 (60)	2 (40)	
**Usa mais de cinco remédios**				
Sim	80 (40)	58 (72,5)	22 (27,5)	0,135
Não	120 (60)	72 (60)	48 (40)	
**Esquece de tomar remédios**				
Sim	28 (14,1)	20 (71,4)	8 (28,6)	0,430
Não	171 (85,9)	109 (63,7)	62 (36,3)	
**Perdeu peso recentemente**				
Sim	61 (30,7)	42 (68,9)	19 (31,1)	0,429
Não	138 (69,3)	87 (63)	51 (37)	
**Frequentemente se sente triste**				
Sim	33 (16,6)	23 (69,3)	10 (30,3)	0,331
Não	165 (82,9)	106 (64,2)	59 (35,8)	
**Tem incontinência urinária**				
Sim	28 (14,1)	22 (78,6)	6 (21,4)	0,100
Não	171 (85,9)	107 (62,6)	64 (37,4)	
**Tempo de deslocamento (três metros)**				
0-10 segundos	56 (28)	29 (51,8)	27 (48,2)	
11-20 segundos	86 (43)	55 (42,3)	31 (44,3)	**0,008**
>20 segundos	58 (29)	46 (79,3)	12 (20,7)	

Avaliando os aspectos que indicam a fragilidade, dois apresentaram associação estatística: internação nos últimos 12 meses e desempenho funcional. Foi verificada associação entre queda e número de internações nos últimos 12 meses (p=0,046), sendo que 81% dos idosos que internaram de uma a duas vezes nesse período apresentaram o evento de queda.

O desempenho funcional foi avaliado pelo teste “levante e ande” cronometrado, no qual o participante deveria levantar-se da cadeira, caminhar três metros e retornar ao ponto de origem, sentando-se novamente. Nessa avaliação, a Tabela 2 mostra que houve correlação estatística positiva com o número de queda, e quanto maior o tempo de deslocamento, maior o número de quedas (p=0,008; rho=0,195). No teste qui-quadrado, também foi evidenciada associação com quedas (p=0,008).

**Tabela 2:** Atividades básicas diárias e associação com quedas (N=200), Juiz de Fora, Minas Gerais, Brasil, 2019

Atividade		Quedas n (%)		Valor de *p*
	Frequência	Sim	Não	
**Banho**				
Independente	185 (92,5)	116 (62,7)	69 (37,3)	
Alguma dependência	11 (5,5)	11 (100)	0 (0)	**0,038**
Dependente	4 (2)	3 (75)	1 (25)	
**Vestir-se**				
Independente	188 (94)	120 (63,8)	68 (36,2)	
Alguma dependência	4 (2)	4 (100)	0 (0)	0,270
Dependente	8 (4)	6 (75)	2 (25)	
**Higiene pessoal**				
Independente	193 (96,5)	124 (64,2)	69 (35,8)	
Alguma dependência	6 (3)	6 (100)	0 (0)	0,077
Dependente	1 (0,5)	0 (0)	1 (100)	
**Sentar, deitar e levantar**				
Independente	191 (95,5)	121 (63,4)	70 (36,6)	0,028^*^
Alguma dependência	9 (4,5)	9 (100)	0 (0)	
Dependente	0 (0)	0 (0)	0 (0)	
**Controle esfincteriano**				
Completo	176 (88)	111 (63,1)	65 (36,9)	
Acidentes ocasionais	21 (10,5)	16 (76,2)	5 (23,8)	0,217
Incontinente	3 (1,5)	3 (100)	0 (0)	
**Alimentação**				
Independente	197 (100)	128 (65)	69 (35)	
Alguma dependência	3 (1,5)	2 (66,7)	1 (33,3)	0,951
Dependente	0 (0)	0 (0)	0 (0)	

* *Valor de p calculado pelo teste exato de Fisher.*

Na análise bivariada, houve correlação estatística com quedas, e quanto maior o número de quedas, menor a independência (p=0,002; rho=-0,220). As atividades básicas que tiveram associação estatística com quedas foram banho (p=0,038) e a capacidade de se sentar, deitar e levantar (p=0,028). Dos participantes que apresentaram alguma dependência nessas duas atividades, todos tiveram episódio de quedas (Tabela 3).

**Tabela 3:** Capacidade visual e associação com quedas em idosos (N=200), Juiz de Fora, Minas Gerais, Brasil, 2019

Capacidade visual		Quedas n (%)				IC95%	
	Frequência	Sim	Não	Valor de *p*	OR	Inferior	Superior
**Visão de longe**							
Satisfatória	116 (58)	86 (74,1)	30 (25,9)	**0,001**	2,606	1,435	4,733
Com perda	84 (42)	44 (52,4)	40 (47,6)				
**Visão de perto**							
Satisfatória	132 (68)	88 (66,7)	44 (33,3)	0,464	-		
Com perda	62 (32)	38 (61,3)	24 (38,7)				

*IC95% - Intervalo de Confiança de 95%; OR - Odds Ratio.*

Na avaliação da capacidade visual de longe, 58% dos idosos possuíam visão satisfatória, e 42% possuíam perda moderada a severa. Na análise bivariada, a acuidade visual para longe apresentou significância quando associada à queda (p=0,001). A correlação de Spearman confirmou esse achado em que quanto maior a perda da visão, maior o número de quedas (p<0,001; rho=-0,248). Idosos com perda na acuidade visual para longe apresentaram uma chance 2,606 vezes maior de queda (IC 1,435 - 4,733).

## DISCUSSÃO

Observou-se feminilização da amostra, composta majoritariamente por indivíduos brancos, com ensino fundamental incompleto, católicos, casados ou em união estável, com renda mensal entre um e três salários mínimos e residentes com familiares. Estudo similar identificou que fatores como sexo feminino, baixa escolaridade e viuvez estão relacionados a pessoas com vulnerabilidade^(16)^. Sabe-se que, nessa fase da vida, o apoio da família é essencial na manutenção dos aspectos físicos e psíquicos do idoso, que acumulam perdas, em decorrência da velhice, muitas vezes difíceis de ser enfrentadas. Essa fase da vida exige cuidados específicos, destacando-se o respeito e a promoção da autonomia, cultivando a rede de apoio, sendo esse o caminho para obter cenários futuros de valorização para essa população^(17)^.

Analisando a ocorrência de quedas, 65% dos idosos informaram já ter caído ao menos uma vez; desses, metade apresentava mais de um evento de queda relatado. A ocorrência e recorrência de quedas pode trazer consequências importantes à pessoa idosa, como dependência, isolamento social e perda da capacidade funcional^(18)^. Estudo identificou que, entre as consequências mais prevalentes nos idosos após o evento de quedas, estão as escoriações, as fraturas e os entorses. Além disso, as consequências permanentes se mostram presentes em grande parte dos idosos, como o medo de uma nova queda, prejuízos na capacidade de deambulação, aumento do grau de dependência das atividades diárias com perda da autonomia e surgimento ou agravamento da depressão^(19)^. Estudo que utilizou *design* de jogos para prevenção de quedas evidenciou que o desenvolvimento de um jogo inovador gerou impactos importantes na redução desse agravo^(20)^.

Identificou-se, na amostra, que 14% dos idosos utilizavam instrumentos como andadores e bengalas. Tal fato foi encontrado em estudo semelhante com 214 idosos residentes na comunidade, no qual a prevalência do uso de auxílio para marcha foi de 12,7%^(21)^. Além disso, os comprometimentos físicos decorrentes do avanço da idade, como alteração de postura e equilíbrio, reduzem a eficácia da marcha em idosos, podendo ser indicado nesses casos o uso de dispositivos de auxílio^(22)^.

Destaca-se uma relação significativa entre quedas e déficit cognitivo na amostra, uma vez que quanto menor a capacidade cognitiva, maior o número de quedas. Corroborando esse achado, outro estudo, em que foi analisada a ocorrência de quedas, verificou que o déficit cognitivo manteve associação estatística significativa com esse evento^(23)^. Sabe-se que o declínio cognitivo decorre do processo de envelhecimento e está relacionado a insuficiências na capacidade de concentração e atenção em idosos. Esse fato pode causar também prejuízos na memória curta e levar à distração, o que pode contribuir para a exposição a situações de risco para quedas como tropeços^(24)^. Estudo prévio, realizado no Brasil, evidenciou que a fragilidade está associada a níveis mais baixos e declínios mais acentuados na função cognitiva, com associações mais fortes para a função executiva^(23)^.

Ao realizar a análise da fragilidade, evidenciou-se correlação com as quedas (p<0,001), de modo que quanto mais frágil, mais propenso à queda está o idoso. Resultado semelhante foi verificado em outro estudo desenvolvido com idosos em que a síndrome da fragilidade esteve relacionada ao evento de quedas (p=0,001)^(25)^. Além disso, verificou-se que mais da metade dos participantes não apresentava fragilidade, enquanto outros 46% se apresentaram frágeis em diversos graus. Estudo evidenciou alta prevalência de fragilidade em idosos e ainda destacou a relação de fragilidade com sexo, idade, escolaridade e manifestações motoras^(26)^.

Quanto ao tempo de deslocamento do idoso, verificou-se que ele esteve relacionado à ocorrência de quedas, de forma que 77,7% dos idosos que caíram apresentaram redução na velocidade de marcha. A diminuição da velocidade de marcha é, inclusive, um dos fatores que definem a presença de fragilidade, sendo observada em 100% dos idosos longevos e em 80% dos idosos frágeis em outras pesquisas com essa população^(27)^.

A queda na pessoa idosa pode sinalizar o início de fragilidade ou aparecimento de doença aguda. Dos idosos que apresentaram queda, 26,9% haviam passado por ao menos uma internação hospitalar no último ano. Esses eventos se apresentaram associados estatisticamente (p=0,046). Outra pesquisa corrobora esse achado, na qual 25,3% dos caidores haviam sido internados no mesmo período (p=0,001)^(28)^.

A associação entre queda e internação, embora comum, não foi largamente encontrada na amostra, sinalizando a importância de realizar pesquisas que abordem mais profundamente essa relação. Sabe-se que a fragilidade no idoso o torna mais propenso aos agravos à saúde, o que pode aumentar a necessidade de internação. Após o evento de queda, o idoso frágil precisa de um período prolongado para a sua recuperação e reabilitação, o que pode aumentar o tempo de internação e consequentemente os custos financeiros para o sistema e para o indivíduo. Autores evidenciaram que o rastreamento do risco de quedas deve considerar a ordem e o momento do início da fragilidade física e do comprometimento cognitivo^(27,28)^.

Sobre as atividades básicas diárias, verificou-se que o aumento da dependência nessas atividades esteve relacionado à elevação do número de quedas (p=0,002; rho=-0,220). Pesquisa mostrou que a dependência em atividades básicas se associa às quedas (p=0,049). Todavia, idosos com limitações funcionais podem apresentar falta de equilíbrio ou coordenação motora que torna mais difícil a execução de atividades diárias. Por outro lado, a independência funcional em idosos, especialmente nos longevos, mostrou-se associada à ausência de uma das principais consequências das quedas: a fratura de quadril (p=0,042)^(29)^.

Na análise da acuidade visual de longe, 42% dos idosos apresentaram alguma perda, estando esse fato associado à ocorrência de quedas (p=0,001). Foi verificado também que quanto menor a acuidade visual para longe, maior a ocorrência de quedas (p<0,001; rho=-0,248). A alteração visual no idoso pode gerar dificuldades em manter o controle postural, expondo esse indivíduo à queda. Por outro lado, estudo evidenciou que idosos com redução da acuidade visual possuíam 1,96 vezes mais chance de sofrer uma queda^(30)^. Além disso, identificou-se na amostra que os idosos apresentaram um terço de déficit visual, enquanto 14% relataram perda auditiva. Os autores identificaram que as perdas sensoriais são um fator importante, pois comprometem a capacidade funcional e, portanto, incidem na qualidade de vida dos idosos, diminuindo sua capacidade funcional e tornando-os menos ativos^(31)^.

### Limitações do estudo

O delineamento transversal do estudo constitui uma limitação, uma vez que não permite avaliar relações de causalidade entre as variáveis. Além disso, a amostra foi composta por idosos de uma área específica, com perfil socioeconômico e nível de escolaridade determinados, o que pode limitar a generalização dos achados. Outra limitação relevante é a possível influência da memória dos participantes, que pode comprometer a precisão das informações autodeclaradas sobre quedas e outros eventos retrospectivos.

### Contribuições para a área da enfermagem, saúde ou políticas públicas

Este estudo contribui de forma significativa para o campo da enfermagem ao evidenciar fatores que expõem os idosos às quedas, além de fortalecer a autonomia dos profissionais na implementação de intervenções preventivas seguras e de baixo custo. Em particular, destacam-se a identificação precoce de risco de quedas e a aplicação de instrumentos de avaliação pelos enfermeiros, podendo ser realizada tanto com idosos atendidos na Atenção Primária quanto em instituições de longa permanência. Consideramos que esta é uma intervenção viável, que pode ser amplamente disponibilizada e adaptada a diferentes contextos institucionais.

## CONCLUSÕES

Os fatores identificados como maiores preditores de risco para queda foram sexo, idade, número de comorbidades, equilíbrio e marcha, fragilidade, atividades básicas diárias, e redução da acuidade visual para longe. O reconhecimento dos principais fatores de risco para quedas em idosos, evidenciados no presente estudo, contribui para a reorientação das práticas assistenciais, especialmente da Atenção Primária. Dessa forma, a equipe de saúde pode direcionar a sua prática baseada em evidências e executar suas ações de forma preventiva aos indivíduos com maior risco. Uma prática assistencial mais segura e qualificada possibilita a redução da incidência de quedas e suas complicações.

## Data Availability

Os dados de pesquisa estão disponíveis no corpo do artigo.
